# Revolutionizing Patient Care: A Comprehensive Review of Artificial Intelligence Applications in Anesthesia

**DOI:** 10.7759/cureus.49887

**Published:** 2023-12-04

**Authors:** Amol Singam

**Affiliations:** 1 Critical Care Medicine, Jawaharlal Nehru Medical College, Datta Meghe Institute of Higher Education and Research, Wardha, IND

**Keywords:** future healthcare technologies, ethical considerations, patient care, precision medicine, anesthesia monitoring, artificial intelligence in anesthesia

## Abstract

This review explores the intersection of artificial intelligence (AI) and anesthesia, examining its transformative impact on patient care across various phases. Beginning with a historical overview of anesthesia, we highlight the critical role of technological advancements in ensuring optimal patient outcomes. The emergence of AI in healthcare sets the stage for a comprehensive analysis of its applications in anesthesia. In the preoperative phase, AI facilitates personalized risk assessments and decision support, optimizing anesthesia planning and drug dosage predictions. Moving to the intraoperative phase, we delve into AI's role in monitoring and control through sophisticated anesthesia monitoring and closed-loop systems. Additionally, we discuss the integration of robotics and AI-guided procedures, revolutionizing surgical assistance. Transitioning to the postoperative phase, we explore AI-driven postoperative monitoring, predictive analysis for complications, and the integration of AI into rehabilitation programs and long-term follow-up.

These new applications redefine patient recovery, emphasizing personalized care and proactive interventions. However, the integration of AI in anesthesia poses challenges and ethical considerations. Data security, interpretability, and bias in AI algorithms demand scrutiny. Moreover, the evolving patient-doctor relationship in an AI-driven care landscape requires a delicate balance between efficiency and human touch. Looking forward, we discuss the future directions of AI in anesthesia, anticipating advances in technology and AI algorithms. The integration of AI into routine clinical practice and its potential impact on anesthesia education and training are explored, emphasizing the need for collaboration, education, and ethical guidelines. This review provides a comprehensive overview of AI applications in anesthesia, offering insights into the present landscape, challenges, and future directions. The synthesis of historical perspectives, current applications, and future possibilities underscores the transformative potential of AI in revolutionizing patient care within the dynamic field of anesthesia.

## Introduction and background

Anesthesia, a critical component of medical practice, has evolved significantly over the years, transforming surgical and medical interventions into safer and more sophisticated procedures. The field has witnessed remarkable advancements from the early use of ether in the 19th century to the development of modern anesthetic techniques. Anesthesia plays a pivotal role in ensuring patient comfort, pain management, and, most importantly, the success of various medical interventions [1]. Advancements in anesthesia contribute to patients' comfort during medical procedures and are also integral to improving healthcare outcomes. The ability to tailor anesthesia to individual patient needs, mitigate risks, and enhance recovery has become a hallmark of contemporary medical care. As the healthcare landscape continues to evolve, pursuing innovations in anesthesia becomes paramount for achieving optimal patient care and safety [2].

The emergence of artificial intelligence (AI) has revolutionized the healthcare industry, offering unprecedented opportunities to enhance diagnostics, treatment planning, and patient outcomes. AI has begun to play a transformative role in anesthesia, introducing novel approaches to patient care, monitoring, and decision-making. Integrating AI into anesthesia practices holds the promise of optimizing existing processes and paving the way for entirely new paradigms in healthcare delivery [3].

This comprehensive review explores the intersection of two dynamic fields - anesthesia and AI. By exploring the historical context of anesthesia practices, understanding the importance of continuous advancements in patient care, and scrutinizing the emergent impact of AI in healthcare, this review seeks to examine the current landscape thoroughly. The primary purpose is to synthesize existing knowledge, analyze recent developments, and delineate the potential implications of AI applications in anesthesia. Through this exploration, we aim to shed light on how AI is revolutionizing patient care in the context of anesthesia, offering insights into the challenges, ethical considerations, and future directions.

## Review

Fundamentals of AI in anesthesia

Machine Learning and Deep Learning

Integrating AI in anesthesia hinges upon machine learning (ML) principles and, more specifically, deep learning (DL). ML, a subset of AI, empowers systems to learn from data patterns and experiences, subsequently improving their performance without explicit programming. DL, a sophisticated form of ML, involves neural networks with multiple layers, mimicking the complex neural structures of the human brain [4]. In anesthesia, ML algorithms are trained on diverse datasets encompassing patient demographics, medical history, and responses to anesthesia. These algorithms can discern intricate patterns, facilitating personalized anesthesia planning and prediction of patient outcomes. DL, with its ability to process vast and complex datasets, has shown particular promise in tasks such as real-time monitoring during surgery and predicting optimal drug dosages [5].

Types of AI Algorithms

Various AI algorithms contribute to the multifaceted applications of AI in anesthesia. Classification algorithms, such as support vector machines, are employed for patient risk assessment and outcome prediction tasks. Clustering algorithms, like k-means, aid in grouping patients based on shared characteristics, informing tailored anesthesia approaches [4]. Reinforcement learning, a paradigm where algorithms learn by trial and error, finds application in closed-loop anesthesia systems. These systems continuously adapt anesthesia delivery based on real-time patient responses, ensuring a dynamic and personalized approach to maintaining anesthesia depth. Additionally, natural language processing (NLP) algorithms facilitate extracting valuable information from unstructured clinical notes, contributing to a more comprehensive understanding of patient history and care [6].

Data Acquisition and Processing

The efficacy of AI algorithms in anesthesia is contingent upon the quality and quantity of data available for training and testing. Data acquisition involves collecting a diverse range of information, including patient vitals, medical history, and responses to anesthesia. Electronic health records (EHRs), anesthesia records, and monitoring devices are primary sources that contribute to these datasets [4]. Data processing in the context of AI involves cleaning, preprocessing, and transforming raw data into a format suitable for algorithmic analysis. Feature extraction, a critical step, involves selecting relevant variables from the data that directly influence the algorithm's learning process. Integrating data from disparate sources and developing interoperable systems are vital considerations to ensure the robustness and generalizability of AI applications in anesthesia [7].

Preoperative phase

Patient Risk Assessment

Predictive modeling for patient outcomes: The preoperative phase, a pivotal juncture in the continuum of patient care, emerges as a sphere where AI unveils its transformative capabilities, particularly in the realm of predictive modeling for patient outcomes. At its core, predictive modeling within AI applications represents a powerful tool, utilizing historical patient data to discern and manage potential outcomes. This sophisticated approach hinges on harnessing the wealth of information embedded in patient records, encompassing demographics, medical history, and preexisting conditions [8]. Through the deployment of ML algorithms, these models delve into the intricacies of this multifaceted data, unraveling patterns and relationships that may elude traditional analyses. The outcome is a personalized risk assessment that transcends the boundaries of conventional risk identification. What sets predictive modeling apart is its proactive nature, empowering healthcare providers to anticipate potential complications, tailor treatment strategies, and, ultimately, elevate the standard of patient safety. In this dynamic interplay between data and technology, predictive modeling in the preoperative phase emerges as a beacon of innovation, promising not only to foresee challenges but to actively shape a more optimized and individualized approach to patient care [8].

Preoperative optimization with AI: Preoperative optimization with the aid of AI transcends conventional risk assessment, introducing a paradigm shift in the preparation of patients for surgery. Beyond merely identifying risks, AI algorithms play a crucial role in tailoring preoperative interventions by delving into intricate patient data and considering individualized factors such as comorbidities and lifestyle. This involves the formulation of personalized plans encompassing nutrition, exercise, and medication adjustments to optimize patient health and resilience leading up to surgery [9]. The integration of AI in preoperative optimization represents a proactive strategy aimed at diminishing the likelihood of complications and enhancing postoperative recovery outcomes. Through a comprehensive analysis of patient-specific data, AI facilitates a more nuanced and adaptive approach to preoperative care, heralding a new era of personalized medicine within the surgical domain. This innovative intersection of technology and healthcare promises not only to mitigate risks but also to actively elevate the overall well-being and resilience of patients undergoing surgical procedures [10].

Decision-Support Systems

Anesthesia planning and personalized care: In the preoperative phase, the significance of anesthesia planning is underscored as a pivotal element in patient care, with AI emerging as a transformative force in this arena. AI-driven decision-support systems are now increasingly instrumental in shaping anesthesia plans. Leveraging a spectrum of patient data, encompassing medical history, physiological parameters, and outcomes from predictive modeling, these systems assist anesthesiologists in crafting tailored anesthesia strategies [9]. Through the comprehensive analysis of numerous variables, AI elevates the precision of anesthesia planning, ensuring that the selected approach is intricately aligned with the individualized needs and risks of each patient. This integration of AI in anesthesia planning marks a paradigm shift, moving beyond generalized approaches to embrace a personalized model that holds the promise of enhancing patient safety and optimizing outcomes in the complex landscape of preoperative care [11].

Drug dosage prediction: Accurate drug dosage administration is a cornerstone of safe and effective anesthesia and the integration of AI ushers in a transformative approach to drug dosage prediction. AI algorithms excel in this domain by assimilating a wealth of patient-specific data, understanding pharmacokinetics, and analyzing real-time physiological responses. This predictive capability empowers anesthesiologists with a more precise and personalized strategy for drug administration, significantly reducing the risks associated with under or overdosing. The role of AI in drug dosage prediction not only revolutionizes anesthesiology but also refines drug administration protocols [12]. The adaptive nature of real-time dosage adjustments based on patient responses enhances the anesthesiologist's decision-making process, ultimately improving patient outcomes and fostering a more tailored and optimized anesthesia experience. This intersection of technology and anesthesia exemplifies how AI is not merely augmenting but significantly enhancing the precision and safety of drug administration in the complex and dynamic environment of medical interventions [13].

Intraoperative phase

Monitoring and Control

AI-based anesthesia monitoring: The integration of AI into anesthesia monitoring marks a significant leap forward, introducing a new era of precision and responsiveness in the context of surgical procedures. AI-based anesthesia-monitoring systems harness sophisticated algorithms to interpret real-time physiological data, empowering anesthesiologists with timely and informed decision-making capabilities. Going beyond conventional monitoring methods, these systems provide a dynamic and personalized assessment of a patient's physiological state [14]. By analyzing crucial indicators such as vital signs and drug concentrations, AI algorithms excel at identifying subtle changes that may signify potential complications. This heightened level of awareness enables preemptive interventions, enhancing patient safety and contributing to a more proactive and adaptive approach to anesthesia management. In essence, the incorporation of AI in anesthesia monitoring not only refines the accuracy of real-time assessments but also signifies a paradigm shift towards a more personalized and anticipatory framework in ensuring patient well-being during surgical procedures [15].

Closed-loop anesthesia systems: Closed-loop anesthesia systems mark a transformative shift in the administration of anesthesia, leveraging AI to regulate drug delivery in real-time. These innovative systems operate by continuously monitoring a patient's physiological responses and autonomously adjusting the administration of anesthetic agents to uphold predefined parameters. This closed-loop approach represents a breakthrough, optimizing drug dosages with a keen eye on maintaining a delicate balance and minimizing the risks associated with under or oversedation [16]. The result is a more stable intraoperative environment, contributing to enhanced patient safety and streamlined anesthesia management. The incorporation of AI-driven closed-loop systems not only refines the precision of anesthesia delivery but also allows anesthesiologists to allocate their focus to other critical aspects of patient care. This synergy between technology and healthcare signifies a shift towards a more automated and responsive approach in the intricate field of anesthesia, promising improved outcomes and heightened efficiency in the operating room [17].

Surgical Assistance

Robotics in anesthesia: The integration of robotics in anesthesia represents a groundbreaking synergy between advanced technology and the intricacies of anesthesia delivery. Robotic systems, fortified with AI capabilities, establish precise control over anesthesia administration and monitoring processes. These systems showcase adaptability to the nuanced requirements of diverse surgical procedures, guaranteeing optimal and tailored anesthesia delivery aligned with the specific needs of each patient. The infusion of robotics into anesthesia not only elevates the precision of drug administration but also introduces the capacity for remote monitoring and intervention [18]. This not only expands the horizons of telesurgery but also fosters collaborative healthcare practices, where expertise can be seamlessly shared across geographical distances. The marriage of robotics and AI in anesthesia not only revolutionizes the accuracy of drug delivery but also extends the boundaries of what is achievable in terms of remote healthcare collaboration, ultimately enhancing the quality and accessibility of patient care [19].

AI-guided surgical procedures: AI-guided surgical procedures stand as a momentous stride forward in the intraoperative phase, exerting influence over decision-making processes and elevating the precision of surgical interventions. Within anesthesia, the deployment of AI algorithms involves the analysis of patient data, surgical plans, and real-time feedback. This analytical prowess provides invaluable insights, guiding anesthesiologists in optimizing anesthesia protocols tailored to the specific demands of each surgical scenario [20]. These AI-guided systems play a pivotal role in enhancing the overall efficiency of surgical teams by furnishing real-time recommendations for adjustments, attuned to the evolving conditions of the procedure. Streamlining the anesthesia process, AI-guided surgical procedures contribute substantively to the overarching objective of refining surgical outcomes and expediting patient recovery. In this symbiotic interplay between AI and surgical practice, the potential for improved precision and efficiency and overall advancements in patient care come to the forefront [20].

Postoperative phase

Patient Monitoring and Recovery

AI-driven postoperative monitoring: The postoperative phase is critical in which vigilant monitoring and personalized care are paramount to successful recovery. AI-driven postoperative monitoring systems represent a significant leap forward in providing continuous, real-time assessment of patients as they transition from the operating room to recovery. These systems utilize ML algorithms to analyze a plethora of data, including vital signs, pain levels, and recovery metrics. By doing so, AI facilitates early detection of postoperative complications, enabling healthcare providers to intervene promptly. Integrating AI in postoperative monitoring not only enhances the accuracy of complication detection but also contributes to the efficient allocation of healthcare resources [21].

Predictive analysis for complications: Predictive analysis powered by AI is crucial in anticipating and preventing postoperative complications. ML models trained on extensive datasets can identify patterns and risk factors associated with complications, allowing for the development of personalized risk profiles for patients. This proactive approach enables healthcare providers to implement targeted interventions and preventive measures, minimizing complications during postoperative recovery. AI-driven predictive analysis is thus a valuable tool in improving patient outcomes and optimizing resource utilization in postoperative care settings [22].

Rehabilitation and Follow-Up

AI-based rehabilitation programs: Rehabilitation is an integral aspect of the postoperative phase, influencing the speed and effectiveness of a patient's recovery. AI-based rehabilitation programs leverage sensor technologies and ML algorithms to tailor rehabilitation regimens to individual patient needs. These programs continuously adapt based on real-time patient progress, optimizing rehabilitation. By providing personalized exercises and monitoring rehabilitation adherence, AI contributes to more efficient recovery and improved long-term outcomes. Integrating AI into rehabilitation programs represents a paradigm shift towards precision medicine in postoperative care [23].

Long-term patient follow-up: Ensuring patients' long-term well-being post-surgery involves continuous monitoring and follow-up. AI facilitates this process by automating and enhancing long-term patient follow-up procedures. ML algorithms can analyze postoperative data, patient-reported outcomes, and other relevant information to assess the effectiveness of the intervention over an extended period. AI-driven long-term follow-up enables the early detection of potential issues and supports the development of personalized, extended care plans. This approach contributes to a holistic and patient-centric model of postoperative care, fostering improved outcomes and patient satisfaction [24].

Challenges and ethical considerations

Data Security and Privacy

The integration of AI into anesthesia brings forth a set of challenges concerning data security and privacy that demand meticulous attention. AI systems heavily depend on extensive datasets containing sensitive patient information, making the assurance of the confidentiality and integrity of this data of paramount importance. Concerns such as unauthorized access, data breaches, and the potential misuse of patient information underscore the ethical imperative of implementing AI in anesthesia responsibly. To address these challenges, it becomes imperative to establish robust data security measures. This includes the implementation of advanced data encryption techniques, the enforcement of strict access controls, and the formulation of transparent policies governing data usage. By integrating these safeguards, the healthcare industry can strike a balance, harnessing the potential of AI for improved healthcare outcomes while upholding the principles of patient privacy and data security in the evolving landscape of medical technology [25].

Interpretability and Explainability

The intricate nature of AI algorithms presents a notable challenge in terms of interpretability and explainability, particularly within critical medical domains like anesthesia. Anesthesiologists and healthcare providers must comprehend the decisions made by AI systems in order to trust and validate their recommendations. Achieving transparency in AI models becomes a fundamental necessity to gain acceptance from the medical community. Ethical considerations underscore the importance of AI algorithms providing interpretable outputs, allowing clinicians to understand the rationale behind the decisions made. This transparency not only fosters trust in AI systems but also facilitates collaborative decision-making between these advanced technologies and healthcare professionals. As the integration of AI in anesthesia advances, ensuring interpretability and explainability becomes a cornerstone in bridging the gap between complex algorithms and the human-centric approach vital in medical decision-making [26].

Bias in AI Algorithms

The potential for bias in AI algorithms emerges as a substantial ethical concern, particularly when applied to patient care within the domain of anesthesia. The resulting algorithms may manifest discriminatory behavior if the training datasets used to develop AI models are inherently biased or unrepresentative. This bias has the potential to disproportionately impact certain demographic groups, thereby introducing disparities in the delivery of care. Ethical considerations mandate a thorough and ongoing examination of datasets to identify and mitigate biases, ensuring that AI applications in anesthesia adhere to the principles of fairness and equity in treatment decisions across diverse patient populations. By addressing bias in AI algorithms, the healthcare community can work towards fostering an inclusive and unbiased application of technology in patient care, aligning with the overarching goal of providing equitable healthcare outcomes for all individuals [27].

Patient-Doctor Relationship in AI-Driven Care

The integration of AI in anesthesia prompts contemplation on the evolving dynamics of the patient-doctor relationship. As AI systems play a role in decision-making processes, preserving open communication, empathy, and a human touch in patient care becomes crucial. Ethical considerations revolve around striking a delicate balance between the efficiency and objectivity offered by AI-driven care and the preservation of the empathetic and personalized aspects inherent to the patient-doctor relationship. Ensuring that AI serves as a complement rather than a replacement for human judgment is essential; this approach fosters trust and ensures that patients feel actively engaged in their care. By navigating these considerations thoughtfully, the healthcare community can harness the benefits of AI in enhancing efficiency while upholding the essential human elements that underpin a trusting and collaborative patient-doctor relationship [28].

Future directions

Advances in AI and Technology

The landscape of AI in anesthesia is poised for remarkable advancements as we peer into the future. The anticipation of progress in both AI algorithms and technological innovations foretells a transformative era for anesthesia practices. The evolution of ML and DL techniques promises to yield more sophisticated and context-aware AI models, capable of intricate and nuanced decision-making. As we explore avenues for improvement, the integration of real-time data from an array of sources, including wearable devices and implantable sensors, emerges as a pivotal strategy. This influx of diverse, real-time information holds the potential to significantly enhance the accuracy and responsiveness of AI-driven anesthesia systems. Furthermore, the horizon of possibilities widens with the ongoing developments in edge computing and cloud technologies. These technological advancements are expected to streamline the integration of advanced AI capabilities into anesthesia practices, paving the way for more dynamic and adaptive decision-support systems. In this era of continuous innovation, the convergence of cutting-edge AI algorithms and technological infrastructure is set to redefine the standards of precision and efficiency in anesthesia delivery [29].

Integration of AI into Routine Clinical Practice

The trajectory of AI in anesthesia unmistakably leads towards a future where integration into routine clinical practice is not only likely but essential. AI-driven decision-support systems, monitoring tools, and closed-loop anesthesia systems are poised to evolve into standard components of anesthesia protocols. This shift holds the promise of not only streamlining workflow within healthcare settings but also significantly enhancing the efficiency and precision of anesthesia delivery. However, the successful integration of AI into routine clinical practice necessitates a collaborative effort among technology developers, healthcare providers, and regulatory bodies. Establishing standardized guidelines becomes paramount, ensuring a unified approach to the incorporation of AI in anesthesia. Addressing safety concerns is equally critical, warranting a meticulous evaluation of AI-driven systems to uphold patient well-being. Moreover, achieving seamless interoperability with existing healthcare infrastructure is crucial for a harmonious amalgamation of AI into routine clinical practices. As we embark on this transformative journey, fostering collaboration and adherence to robust standards will be pivotal to realizing the full potential of AI in reshaping the landscape of routine anesthesia care [13].

Potential Impact on Anesthesia Education and Training

The integration of AI into anesthesia practice is poised to bring about a profound transformation in education and training within the field. Anesthesia training programs are likely to undergo adaptation to incorporate comprehensive AI education, ensuring that future anesthesiologists are equipped with the skills to effectively leverage AI tools in their practice. AI-powered simulation technologies could play a pivotal role in this transformation by providing realistic training scenarios, allowing practitioners to refine their skills in managing AI-driven systems in a controlled environment [30]. Continuous education efforts will be imperative to keep healthcare professionals abreast of the latest advancements in AI, ethical considerations, and best practices in utilizing AI for anesthesia. The dynamic nature of AI technology requires a commitment to ongoing learning, ensuring that healthcare professionals remain well-informed and adept at integrating these innovations into their daily practice. As the field of anesthesia evolves with the integration of AI, providing education and training will be key in preparing the next generation of anesthesiologists for a future where technology is seamlessly woven into the fabric of patient care [15].

## Conclusions

This comprehensive review highlights the transformative impact of AI on the landscape of anesthesia, spanning the preoperative, intraoperative, and postoperative phases. The integration of AI algorithms has demonstrated its potential to enhance patient care through personalized risk assessments, optimized drug delivery, and advanced monitoring systems. Looking forward, the future of AI in anesthesia holds great promise, marked by anticipated advances in technology and ML. These innovations are expected to increase precision, adaptability, and patient-centricity in anesthesia delivery. However, as we embrace this future, navigating challenges related to data security, interpretability, and the evolving dynamics of the patient-doctor relationship is essential. The implications for patient care are profound, with the potential for improved outcomes, reduced complications, and heightened overall satisfaction. As healthcare systems evolve to accommodate these advancements, collaborative efforts between healthcare professionals, technology developers, and policymakers will be crucial to ensuring the responsible integration of AI and unlocking its full potential in shaping the future of anesthesia.
